# Functional Protein-Based Bioinspired Nanomaterials: From Coupled Proteins, Synthetic Approaches, Nanostructures to Applications

**DOI:** 10.3390/ijms20123054

**Published:** 2019-06-22

**Authors:** Dong Zhang, Yi Wang

**Affiliations:** 1 National Engineering Laboratory of Intelligent Food Technology and Equipment, Key Laboratory for Agro-Products Postharvest Handling of Ministry of Agriculture, Key Laboratory for Agro-Products Nutritional Evaluation of Ministry of Agriculture, Zhejiang Key Laboratory for Agro-Food Processing, Fuli Institute of Food Science, College of Biosystems Engineering and Food Science, Zhejiang University, Hangzhou 310058, China; dorothy.zhang@connect.polyu.hk; 2 Department of Applied Biology and Chemical Technology, The Hong Kong Polytechnic University, Hong Hum, Kowloon 999077, Hong Kong; 3 State Key Laboratory of Chinese Medicine and Molecular Pharmacology (Incubation) and Shenzhen Key Laboratory of Food Biological Safety Control, Shenzhen Research Institute of Hong Kong Polytechnic University, Shenzhen 518057, China

**Keywords:** bioinspired, nanomaterials, functional protein, tissue engineering, drug delivery, water purification

## Abstract

Protein-based bioinspired nanomaterials (PBNs) combines the advantage of the size, shape, and surface chemistry of nanomaterials, the morphology and functions of natural materials, and the physical and chemical properties of various proteins. Recently, there are many exciting developments on biomimetic nanomaterials using proteins for different applications including, tissue engineering, drug delivery, diagnosis and therapy, smart materials and structures, and water collection and separation. Protein-based biomaterials with high biocompatibility and biodegradability could be modified to obtain the healing effects of natural organisms after injury by mimicking the extracellular matrix. For cancer and other diseases that are difficult to cure now, new therapeutic methods involving different kinds of biomaterials are studied. The nanomaterials with surface modification, which can achieve high drug loading, can be used as drug carriers to enhance target and trigger deliveries. For environment protection and the sustainability of the world, protein-based nanomaterials are also applied for water treatment. A wide range of contaminants from natural water source, such as organic dyes, oil substances, and multiple heavy ions, could be absorbed by protein-based nanomaterials. This review summarizes the formation and application of functional PBNs, and the details of their nanostructures, the proteins involved, and the synthetic approaches are addressed.

## 1. Introduction

With billions of years of evolution, natural organisms have developed highly effective biological mechanisms to form surfaces with exclusive or exceptional characteristics. Recently, the development of bioinspired materials relied firstly on finding the manifestation of the structures and physicochemical properties of such mechanism, followed by production and synthesis of materials that reproduced a similar effect [1]. Nanomaterials, because of their size and topology, were believed to have enhanced permeability and retention (EPR) effect, which enable them to gain more access to cellular and tissue compartments. The size, shape, and surface chemistry of nanomaterials could be readily programmed by physiochemical technology, that might contribute to the potency of nanomaterials to form various structures, including particles, fibers, and porous sponges, which can be used as carriers and scaffolds for application in the medicine and health fields [2]. Along this line, combining with bioinspired knowledge and nanomaterial technology, bioinspired nanomaterials have gained much attention due to their promising prospect in life science studies [3].

Therefore, in the process, biomimetic nanomaterials have spurred efforts to mimic the morphology and functions of natural materials, and developed new generations of bioinspired nanomaterials, including bacteria-inspired, virus-inspired, fungus-inspired, and mammalian cell-inspired nanosystems. The popular material systems with nanostructures include, lipid-based system, polysaccharide-based system, protein-based nanostructure system, and hydrogel-based system. Of which, protein-based bioinspired nanomaterials (PBNs), which combine the advantage of the physical properties, stability, prolonged control release, and triggering effect, have recently shown much effects on the realization of a vast range of specific functional materials and structures. Compared with cell- or bacteria-involved nanomaterials, PBNs proposed fewer biological barriers such as opsonization, immune clearance, negotiation with vascular systems, and better efficiency and safety in clinical usage. Depending on the complicated three-dimensional (3D) molecular structures and unique sequence properties, PBNs have been widely used in medicine, biological engineering, environmental engineering, electronics, and information technology.

**Figure 1:**
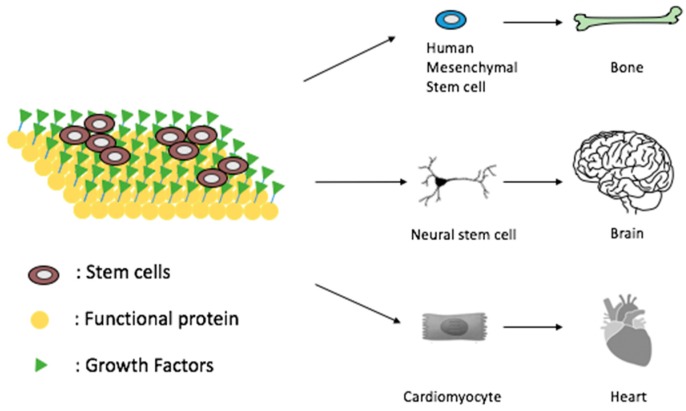
Protein biomimetic materials for tissue regeneration.

The major two categories of PBNs are nanoparticles and nanofibers, which have wide applications in drug delivery and tissue regeneration, as well as in environmental engineering. Nanoparticle-based drug delivery vehicles, such as calcium phosphate nanoparticles, gold nanoparticles and nanodiamonds have been demonstrated to have unique advantages in treating bone diseases through stimulating mineralization or promoting bone cell activity. For precision drug delivery, PBNs could specifically release drugs into the target area based on the pH gradients, redox potentials, and selective enzyme reactions. Certain destruction mechanisms, such as pH-sensitive lipid and protein combined self-assembled structure, particular hydrophobic interaction of the cargo, and direct targeting receptor trigger identified the drug release of PBNs [4,5]. PBN nanofibers could mimic the extracellular matrix (ECM) of native tissues, the ideal scaffolds structure and physical properties for new tissue regeneration [6,7,8]. Schematic diagram of stem cell based tissue regenerations using protein biomimetic materials is shown in Figure 1. The molecular structure and interaction of the proteins, the size and porosity of the nanofibers, and the microenvironment provide integrity, biocompatibility, and slow biodegradation rate for tissue regeneration in vitro and in vivo. Encapsulation of the drug-loaded stimuli-responsive nanoparticles into bioinspired nanofibers would successfully manipulate the local environment to control cell growth and differentiation for tissue regeneration [9,10]. Polymer nanofiber membranes were the commonly studied materials for water filtration applications [11]. The size and the ion affinity of the nanomembranes could contribute a lot in various applications in environmental engineering.

Therefore, due to different physical and chemical properties, for instance, size, charge, hydrophilicity, structure and composition, PBNs have various applications in tissue regeneration (Table 1), drug delivery, and other biological activities as well as water purification in environmental engineering. However, the relationship between the structure of PBNs and its application still needs further exploration. In this review, we summarize the functional PBNs and their potential applications and limitations based on current literature sources.

## 2. Biomimetic Materials for Tissue Regeneration

### 2.1. Bone Regeneration

#### 2.1.1. Collagen-Based Nanomaterials

Collagen, the most abundant protein in human body, is good for cell adhesion, proliferation, and differentiation. It was widely used in tissue engineering studies. Chitosan [43], hydroxyapatite, and alendronate [44], respectively, were coupled with collagen to form scaffolds for orthopedic tissue repair. The scaffold displayed a larger pore size, higher percentage porosity, higher compressive modulus, and a greater capacity for water uptake. The relevant features of the scaffold allowed faster mesenchymal stem cell (MSC) proliferation and differentiation [43,44]. 

Inspired from the bone extracellular matrix, a novel proteinaceous hybrid matrix was obtained by fusing osteocalcin–fibronectin with collagen. After fabrication, the biomaterial preserved the capacity to promote stem cell adhesion, and the structural stability was improved to more than one month. And the results showed that it could improve the in vivo bone regeneration of calvarial defects to over six weeks [8]. 

Four doses of the synthetic P24 peptide (24 amino acids), which were derived from the bone morphogenic protein-2 (BMP-2), were loaded into the nano-hydroxyapatite/recombinant human-like collagen/poly (lactic acid) composite scaffolds, respectively. The in vivo experiments revealed that the scaffolds with P24 peptide significantly increased the osteo-induction and bone regeneration in a dose-dependent manner in rats [12]. 

Biphasic calcium phosphate nanoparticles and collagen were composited to make a scaffold matrix with good biocompatibility. It could control the release of the dexamethasone, and promote the osteogenic differentiation of human MSC. In vivo experiment showed that this novel scaffold accelerated the ectopic bone tissue regeneration at the dorsa of athymic nude mice [45]. 

#### 2.1.2. Serum Albumin-Based Nanomaterial

It was known that the coating of serum albumin supports stem cell attachment and proliferation; thus serum albumin-coated biomaterials can be used as vehicles for cell transplantation [46]. It was reported that two kinds of bovine serum albumin (BSA)-based nanomaterials were fabricated: 1) BSA–Ag nanoparticles with octacalcuim phosphate and graphene oxide/chitosan; 2) penta-peptide glycine–arginine–glycine–aspartate–serine grafted BSA films. The results illustrated that both of the two biomaterials significantly promoted osteoblastic progression and enhanced biocompatibility [13]. 

#### 2.1.3. Zein-Based Nanomaterials

Zein is insoluble in water due to its specific amino acid sequence. Zein could be used as a mineralization template for the growth of calcium phosphate. The mechanical strength-enhanced film served as a friendly environment for the attachment, spread, and proliferation of fibroblast cells, which suggested that the zein-based film could serve as a biomimetic scaffold for bone regeneration [14]. Other than serving as a film, a biomimetic zein polydopamine-based nanofiber also demonstrated its potential in bone regeneration. First, bone morphogenic protein-2 (BMP-2) was coupled to TiO_2_ nanoparticles, which could extend the retention time at the target areas. Then, the fabrication of zein polydopamine and TiO_2_ nanoparticles with BMP-2 was undergone by electrospinning. The nanofibers improved the adhesion, mineralization, and differentiation of fetal osteoblast cells [15].

#### 2.1.4. Silk-Based Nanomaterials

Silk-derived protein was also used for bone regeneration applications. Biphasic silk fibroin scaffolds were formed and it was showed that, with integrated anisotropic and isotropic functionalized heparin, the scaffold could increase the expression of tendon/ligament markers and the content of collagen I protein. With the delivery of both TGF-β2 and GDF5, the scaffold with isotropic porosity upregulated the expression of the cartilage markers and increased the content of the collagen II protein [16]. 

The biomimetic nanofibrous scaffold composited of the silk fibroin and carboxymethyl cellulose was formed. And the higher contact angle and water uptake capacity of the fibroin/cellulose scaffolds presented the superior cell-supporting property compared to pure silk fibroin scaffolds. By the means of biomineralization into nuclear bioactive calcium phosphate, the scaffold promoted osteogenic differentiation [17]. 

The silk sericin from the *Antheraea pernyi* silkworm contained a lower percentage of serine and tyrosine, and was hydrophilic. As a novel nanofiber, it was used to evaluate the capability of hydroxyapatite crystal nucleation. The results suggested that this bioinspired nanofiber stimulated the adhesion and proliferation of the bone MSC [18]. 

### 2.2. Cardiac Cells Regeneration

Myocardial cells do not regenerate after infarction, so myocardial infarction (MI) is the major cause of mortality. Scaffolds are helpful for cardiac tissue regeneration to treat MI and avoid mortality.

#### Silk Fibroin-Based Nanomaterials

The silk fibroin isolated from non-mulberry Indian tropical tassar silkworm was used for the fabrication of the patterned silk film to develop isotropic and anisotropic scaffolds to treat MI. It was showed that silk fibroin film could promote the formation of the 3D silk cell monolayers, which help in the regeneration of cardiac tissue [19,21]. 

Proteins from the silk cocoons of yellow hornets were dissolved in concentrated salt solutions. The films were formed by casting and the mechanical properties of the films were evaluated. Among the four selected proteins, the films of Vssik1 and Vssik2 showed significant improvement on cell adhesion [20].

Human bone MSCs were transplanted on the poly (glycerol sebacate)/fibrinogen/Vascular endothelial growth factor (PGS/Fib/VEGF) scaffolds. The immunohistochemistry results indicated that the cardiac marker proteins troponin and actinin, and endothelial cell marker protein CD31 were expressed in the transplanted scaffolds. The PGS/Fib/VEGF scaffolds facilitated the differentiation of the MSCs into cardiac cells and endothelial cells [22]. 

### 2.3. Corneal Tissue Regeneration

Corneal blindness accounts for nearly 10 million cases of vision loss worldwide [47]. Currently, the production of a fully functional corneal construct is still not successful. Using biomaterial-based scaffolds to support the cell proliferation and differentiation to repair diseased or damaged cornea endothelial cells will be a potential solution for the formation of the functional corneal construct. 

#### Silk-Based Nanomaterials

The advantages of silk-based film include transparency, mechanical integrity, biocompatibility, and slow biodegradation. So far, it has been demonstrated that silk films can successfully promote the adhesion and proliferation of MSCs. However, to support the growth of both corneal fibroblast and endothelial cells, which is necessary for corneal tissue regeneration, remains a challenge. The fusion of silk materials and the inherent optical clarity provides a new approach to generate useful films [23].

Silk biomaterials were bio-functionalized with arginylglycylaspartic acid (RGD), the tripeptide Arg–Gly–Asp within fibronectin that mediates cell attachment. The RGD-modified silk surface could enhance the cell attachment, proliferation, alignment, and expression of collagens and proteoglycans. This porous and transparent RGD-coupled silk protein scaffold demonstrated a strategy for corneal tissue engineering [24].

Films with different thicknesses were obtained by casting a mixed solution of silk fibroin and polyethylene oxide on the polydimethylsiloxane (PDMS) substrates. Human and rabbit corneal fibroblasts were seeded onto the films, and the amount of the characteristic proteins of the cornea cells were measured. The results showed that the 2 μm film had the optimal promotion of the adhesion and proliferation of both human and rabbit corneal fibroblasts [23].

To maintain the specific functions of the corneal endothelial cells (CEnCs), β-Carotene (β-C) was blended with silk fibroin (SF) to form a scaffold. Compared to pristine SF scaffold, the β-C/SF scaffold showed enhanced mechanical properties, hydrophilicity, and transparency. Exclusively, the β-C in the scaffold enhanced the ATPase pump function of the CEnCs. The results indicated that the β-C/SF scaffold might be the suitable alternative corneal endothelium substitute for the transplantation [25].

Another biomaterial used for transplantation was prepared by adding lysophosphatidic acid (LPA) to SF. The LPA in the scaffold would be released in the injured cornea. It was reported that the scaffold facilitates the growth of fibroblasts, keratinocytes, and endothelial cells, while greatly improving the various properties of the original SF films. The expression of the specific genes and proteins of the cornea endothelial cells confirmed the corneal tissue regeneration in the LPA/SF scaffold [26].

### 2.4. Chondrogenesis Tissue Regeneration

#### Albumin-Based Nanomaterial

Gelatin is a protein produced by partial hydrolysis of the collagens that were extracted from the skin, bones, cartilage, and ligaments. The structure and biocompatibility of gelatin has led to its use in a wide range of biomedical applications. A protein-based 3D porous scaffold was fabricated by blending gelatin and albumin. Meanwhile, two growth factors, TGFβ3 and TGFβ2, respectively, were added to the scaffold. The capacity of chondrogenic differentiation of the scaffolds was measured. The result showed that TGFβ3-coupled nanoparticle provided a suitable environment for the chondrocytes culture [27,28].

### 2.5. Vascular Tissue Regeneration

Therapies aiming at the vascular tissue regeneration have been studied to relieve the symptoms of ischemia and prevent organ damage due to hypoxia, reperfusion, or capillary leak. However, gene therapies or small molecular approaches have largely failed. Biomaterial assisted therapy, which could promote the functions and regeneration of vascular tissues, were widely studied and could be of potential for the treatment of vascular diseases [48].

#### 2.5.1. Elastin-Based Nanomaterial

Recombinant elastin-like polymers, because of the property of mimicking ECM, have been studied for drug delivery applications and for the repair of damaged elastic tissues. The recombinant human elastin-like polypeptides (HELPs) were obtained by the insertion of cross-linking sites of glutamine and lysine in the recombinant polymer. The HELPs showed improved mechanical strength. The cytotoxicity tests on the human umbilical vein endothelial cells were also performed [29,30].

#### 2.5.2. Fibrin-Based Nanomaterial

Recently, the relevant experiments successfully demonstrated that 3D fibrin nanomaterials-based microenvironment showed the ability of angiogenesis promotion by stimulating the related growth factors, such as TGF-β1. TGF-β1 could promote the differentiation of MSC to the myogenic lineage and enhance mechanical properties of the vascular constructs. The results revealed that the conjugation of TGF-β1 in a 3D fibrin matrix induced the activated Smad2 signal in fibrin-embedded cells for several days, leading to an increased vascular contractility [31,32].

#### 2.5.3. Silk-Based Nanomaterial

Meanwhile, recent study showed that, as scaffolds, silk-based biomimetic materials could be used to promote angiogenesis. *Antheraea assama,* silk-based non-woven β-sheet fibroin possessed characteristics of a pore size of 150 µm, a porosity of 90%, and a water uptake capacity of 85%. The fibroin exhibited satisfied blood compatibility, excellent human cell attachment, and better spreading and migration ability. It also improved the synthesis of nitric oxide to promote angiogenesis [33,34].

### 2.6. Neuroregeneration

Neural regenerative medicine is a promising strategy for the repair or replacement of damaged central nervous system, including brain. In this field, PBNs with viscoelastic properties showed potential activity in nerve regeneration [35,37].

#### 2.6.1. Fibrin-Based Nanomaterial

A 3D nanofiber hydrogel with hierarchically aligned fibrins was prepared by electrospinning and molecular self-assembly. The Schwann cells (SCs) and dorsal root ganglions (DRGs) were cultured on the fibrin hydrogels and transplanted to bridge the nerve defects in rats. The results confirmed that the fibrin-based hydrogel could promote the proliferation and migration of SCs and the regrowth of axonal [36].

#### 2.6.2. Chicken Albumin-Based Nanomaterial

Natural chicken albumen, which had high proton conductivity, was used as the coupling electrolyte film for the fabrication of the organic/inorganic hybrid synaptic devices. The albumin-based synaptic devices successfully functioned in the paired-pulse facilitation, dynamic filtering, and short-term to long-term memory transition [38].

### 2.7. Skin and Muscle Regeneration

#### 2.7.1. Elastin-Based Nanomaterials

Mimicking the physicochemical properties of the native skin was essential for the treatment of the skin injures. Elastin, as a structural protein, is one of the main constituents of the skin ECM. It owns natural biocompatible features, and was widely used in tissue engineering [39].

It was reported that the scaffold containing gelatin, cellulose acetate, and elastin was fabricated by electrospinning, which promoted the attachment and proliferation of the fibroblast cells [40].

Many cellular responses are triggered by cellular recognition of the surface features of the biomaterials, and lead to the signal transduction cascades. HELPs, which could be specifically recognized by cells, have been successfully applied as surface coating proteins. Two kinds of HELPs, as functional proteins, were placed on the surface of the biomaterials, and the coated biomimetic materials improved the proliferation and differentiation of H9c2 myoblast [41,42].

#### 2.7.2. Collagen-Based Nanomaterials

Tilapia skin collagen sponge, a marine collagen, has attracted attention because of its abundance, similarity in structure with the human skin collagen, and low price. Tilapia skin collagen sponge and the original electrospun collagen nanofibers were fabricated by electrospinning, and the ability to induce skin regeneration was examined both in vitro and in vivo [49]. To prevent the infection from the exposed wound, bioactive glass (BG) with antibacterial potential, was incorporated into the tilapia skin collagen (TSC) via electrospinning. Human keratinocytes, human dermal fibroblasts, and endothelial cells were selected to study the usage of the TSC/BG nanofibers. In vivo experiments were consistent with the results demonstrated in vitro; the TSC/BG nanofibers showed faster skin regeneration [50]. Another strategy to functionalize TSC was the fabrication of different molecular weights of chitooligosaccharides (COSs), sodium alginate (SA), and TSC with 1-ethyl-3-(3-dimethylaminopropyl)carbodiimide hydrochloride as a cross-linking agent. Relevant in vitro experiments showed that the compound with various beneficial features could mimic the environment of skin tissue [51].

Chitosan (CS) was also used to mix with collagen for the formation of biomaterials. First, the positively charged CS blended with the negatively charged type I collagen by layer-by-layer self-assembly as coating bilayers. The coating bilayers were conjugated with the nanofibrous matrices, which were co-electrospinned with polycaprolactone (PCL)/cellulose acetate (CA). The modification of PLC/CA nanofibrous matrices showed higher wound recovery rate and degradation rate than that of the initial nanofibrous matrices [52].

### 2.8. Dental Tissue Regeneration

Dental caries, as a usual prevalent infectious disease, bothered more than half of the population around the world. Traditional root canal therapy leaves patients with the loss of tooth sensitivity and makes them susceptible to secondary infections. Therefore, the utilization of pulp ECM in bioengineering possessed great potential for the enhancement of the limited regeneration capacity of the tissue [53].

#### 2.8.1. Silk-Based Nanomaterials

The potential of using silk fibroin-based scaffolds for pulp regeneration was examined by checking the growth of the dental pulp stem cells (DPSCs) planted on the materials. The basic fibroblast growth factor (bFGF), as a signaling molecule inducing the tissue regeneration, was fabricated with the silk fibroin scaffold via lyophilization. The growth of the planted DPSCs could be promoted by bFGF. The dentin-like tissue formation and vascular distribution were observed in the new generated tissue [54].

#### 2.8.2. Collagen-Based Nanomaterial

DPSCs were cultured on six various biomaterials. After 12 weeks, the differentiation and growth of the cells were analyzed by radiographic, histological, and immunohistochemical evaluations. Dentin matrix protein 1 (DMP1), a noncollagenous extracellular matrix protein, has been proven as a signal factor to promote the transformation of DPSCs to odontoblast-like cells. Thus, DMP1 was impregnated on the collagen scaffold, and the strong antidentin sialoprotein signals of the newly regenerated dentin were detected. This implied that the constituents of DPSCs, DMP1, and collagen scaffolds were useful to repair the endodontic perforations [55].

To provide optimal cellular proliferation, migration, and a differentiation matrix for specific stem cells, complex ECM will be simulated. The nanomaterials were blended together with the type I collagen and chitosan at a ratio of 1:1, which would facilitate the hydroxyapatite nucleation. DPSCs and periodontal ligament stem cells (PDLSCs) were respectively planted onto this nanomaterial with over 20 growth factors. The results indicated that the presence of three proteins, DMP1, desmoplakin, and protein decapentaplegic were critical to provide biological activity of cell differentiation [56].

## 3. Biomimetic Material to Enhance the Mechanical Properties of Films, Such as Tensile Strength and Flexibility

Generally, films formed by naturally derived proteins have disadvantages in appearance and mechanical properties, such as transparency, elongation, and tensile strength. By inserting functional groups into the protein-based materials, intensified characteristics such as high tensile strength, strong flexibility, and large coverage area would be acquired.

### 3.1. BSA-Based Films

Protein-based nanoporous films have numerous potential applications in environmental engineering and biomedical engineering. Inspired by the bacterial biofilms, the BSA-based film was produced through a combined process of protein fibrillation and reverse dialysis of BSA. The films were free-standing, biodegradable, and nontoxic, with tunable thickness, high transparency, and remarkable endurance in various solutions. Similar to the amyloid nanofibril-based materials, the BSA films showed great adsorptions of diverse materials, including nanomaterials, organic dyes, heavy-metal ions, and enzymes [57].

### 3.2. Soy-Based Composite Films

Generally, materials based on renewable resources showed inferior mechanical and physicochemical properties, because of the high contents of hydrophilic groups. Soluble soybean polysaccharide (SSPS) as the matrix, and catechol-functionalized soy protein isolate (SPI-CH) as the adhesive component were combined and conjugated. The result polymer showed remarkable enhancements for mechanical properties. The tensile strength increased from 2.80 MPa of the unmodified SPI film to 4.04 MPa of the modified one, which was a 44% improvement [58].

Poly(dopamine) (PDA)-modified microcrystalline celluloses (MCC) was combined with SPI to improve the tensile strength. PDA, the adhesive layer, could be coated on MCC to form PDMCC. Because of the interfacial adhesion between PDMCC and SPI, the tensile strength was improved by 82.3% from the SPI film [59].

Comparing SPI-CH combined with SSPS, and SPI combined with PDMCC, a higher improvement on the tensile strength from unmodified SPI film was found by using PDMCC.

### 3.3. Squid Sucker Ring Teeth Proteins-Based Films

The proteins from squid sucker ring teeth were widely used in the fabrication of photo-cross-linked films, which exceeded the mechanical properties of most natural and synthetic polymer films. Recombinant squid sucker ring teeth proteins were engineered into a range of structural and functional materials, including nanopatterned surfaces and photo-cross-linked films. Engineering of new materials inspired by biological structures facilitates the molecular characterization of natural materials and the effective translation of their molecular designs into a wide range of bioinspired materials [60].

### 3.4. Chicken Egg White (CEW)-Based Films

Spontaneously, 5,6-dihydroxyindole (DHI) would easily convert CEW, which originally was a matrix, into insoluble hydrogels by simple heating. A black, water-soluble, and processable artificial biomelanin (ABM) film can be produced and representationally used in electronics and biomedical applications [61].

### 3.5. Silk-Based Films

Inspired by the features of visual fluoroscopy and composite hybridization of the chitin/protein complex of arthropod cuticle, silk fibroin were considered to composite with chitin for film productions. The molecular-level dimensional conformity motivated the connection of chitin nanofibers and silk fibroin. The hybrid material demonstrated its utility as a functional/structural component for emerging applications in electronic and wearable devices, such as smart contact lenses [62].

To further improve the flexibility of the silk fibroin film, a plasticizer, such as glycerol, was dissolved in water and blended with silk fibroin for further reactions. Glycerol appeared to replace water in silk fibroin chain hydrationthus, leading to the initial stabilization of the helical structures in the films [63].

### 3.6. Bitter Vetch Protein-Based Films

Bitter vetch protein was used for film productions. The film containing positively charged spermidine, alone or with low amounts of glycerol, showed high tensile strength. The spermidine serves as both primary and secondary plasticizers. It ionically interacts with the proteins and facilitates the reduction of the intermolecular forces along the protein chains, consequently improving the film flexibility and extensibility. Confirmed by the film permeability tests, both spermidine and low concentration glycerol increased the gas and water vapor barrier properties [64].

## 4. Protein-Based Nanoparticles for Drug Delivery

Protein-based nanospheres are often used as carriers for various kinds of drug delivery, because of their structure, functionality, biocompatibility, biodegradability, and non-toxicity.

### 4.1. Anti-Cancer Drug Delivery

In recent decades, cancer is one of the greatest threats to human health. With many mechanisms of triggered release, such as pH sensitive structure, hydrophobic interaction-based structure, and direct receptor triggered destruction, nanoparticles could perform precise releases of anti-cancer drugs to the cancer cells. Examples were shown in Table 2.

#### 4.1.1. Serum Albumin-Based Nanoparticles

PBNs were applied for the studies of in vitro anti-tumor activity and CT imaging. One nanoparticle was prepared by taking the advantage of the interactions between the gemcitabine (Gem)-loaded gold nanospheres and the bovine serum albumin (Au@BSA). The Au@BSA-Gem showed effective drug delivery to the human pulmonary carcinoma cells [65].

Au was also used in a uniform core-shell gold nanorod/serum albumin (NR@SA) nanoplatform. The structure had less free SA adsorption and higher drug-loading capacity. And it was more sensitive to photoacoustic signals, so it could be used for the detection of cancer cells, which can be applied for cancer diagnosis [66,67,68].

By genetic fusion of an albumin-binding domain and a legumain-substrate peptide with the *trichosanthin* (*TCS*) gene, a new SA-based intelligent hitchhike system, was developed. The anti-tumor activity was evaluated in the animal model for breast cancer and showed obvious improvement compared to the single gene therapy [69,70].

It was reported that a nanoparticle was prepared via bioconjugation of an aliphatic aldehyde-functionalized copolymer to the hydrazine-modified BSA through reversible pyridylhydrazone linkages. In response to the physical, chemical, and biological stimuli, the nanoparticle would undergo the changes of the constitution. Meanwhile, this polymer-protein displayed the characteristics of temperature sensitivity and glutathione (GSH) responsiveness. The encapsulation of doxorubicin (DOX) using the nanoparticles exhibited anti-tumor activities [71,72,73,74].

Another BSA-based nanoparticle was acquired by the surface modification of BSA using histamine through amide reaction. The nanoparticle was effective in the treatment of breast cancer in both the in vitro and in vivo experiments. And in particular, glycol-transferrin-inspired nanoparticles bypassed and decreased the P-glycoprotein-mediated drug efflux and led to more effective treatment of multidrug-resistant breast cancer compared to free drugs [75,76].

#### 4.1.2. Silk-Based Nanoparticles

Silk sericin, a glue-like protein of the wild silkworm, was used to encapsulate hydroxylapatite. It was shown that the silk protein-based nanocarrier was excellent for drug delivery under acidic conditions [77].

#### 4.1.3. Membrane Protein-Based Nanomaterial

Inspired by the biomimetic technologies, the “Emperor Qin’s Terra-Cotta Warriors” were used to design the artificial chimeric exosomes. The nanomaterial was constructed by integrating various cell membrane proteins into synthetic phospholipid bilayers. A hybrid membrane protein CD47 derived from the red blood cells, which plays a key role in evading phagocytosis and increasing the accumulation in tumor tissue, was added to the drug carrier. The modification of the nanomaterial led to higher tumor accumulation and showed better anti-tumor therapeutic effect in the animal model [78].

### 4.2. Drug Delivery for Vascular Diseases

Vascular disorders, including atherosclerotic cardiovascular disease and ischemia/reperfusion injury, cause a range of health problems, which can be severe or prove fatal [79]. Newly developed biomaterials for effective therapy are urgently needed to improve the cure rate of vascular disorders.

A blood brain barrier (BBB)-penetrating nanoparticle was synthesized using albumin, albumin-binding proteins, such as SPARC (secreted protein acidic and rich in cysteine) and glycoprotein 60 (gp60). SPARC and gp60 acted as the functional groups for the penetration through BBB. Paclitaxel (PTX) and fenretinide (4-HPR) were selected and encapsulated by albumin-based nanoparticles, which owned the ability to cross BBB. In addition, the dual delivery of PTX and 4-HPR showed pro-apoptotic effects against the tumor growth in the clinical trials [80].

### 4.3. Drug Delivery for Rheumatoid Arthritis (RA)

Rheumatoid arthritis (RA) is one of the most common chronic autoimmune diseases. In order to decrease the suboptimal response to the therapy, the targeted delivery to the joints using nanoparticles should be applied. The injection of methotrexate-loaded human serum albumin nanomedicines (MTX@HSA NMs) into collagen-induced arthritis (CIA) in mice demonstrated higher drug accumulations and longer retention in the inflamed joints [81].

## 5. Functional Materials—Adhesives

Recently, great progress has been made in the development of the underwater adhesives by using natural materials. Examples were shown in Table 3. However, the development of the interfaces under dynamic turbulence still face great challenges [82,83].

### 5.1. Mussel Proteins-Based Materials

Self-assembly hybrid molecular fibers were obtained from the fusion of mussel foot proteins (Mfps) of *Mytilus galloprovincialis* and CsgA (an amyloidogenic protein that constitutes the major subunit of adhesive curli fibers in *E. coli*) proteins. The genetic fusion of CsgA-Mfp3 and Mfp5-CsgA presented synergistic features of superior adhesion ability and exhibited better tolerance to auto-oxidation at pH ≥ 7. The adhesion energy of the fibers was nearly 20.9 mJ/m^2^, which was 1.5 times greater than the maximum of bio-derived protein-based underwater adhesives that have been reported so far [82].

### 5.2. Serum Protein-Based Materials

Polystyrene (PS) has been widely used for cell culture plates, because it can be easily molded into various shapes and has excellent transparency and physical properties. Recently, a coating system has been developed by binding hydroxyapatite (HAp) to serum protein adsorption layers. After treated by the protein layer, the PS substrate had a large increase in the absorbed amount of human serum albumin (HSA) and human immunoglobulin (Ig) G [84].

### 5.3. BSA-Based Materials

Biomimetic materials with adhesive property could be used to remove dyes. BSA, as both a reductant and a stabilizer, was synthesized on the bifunctional adsorbent-catalytic hemin-graphene. The organic dyes were absorbed on the surface of the material and generated hydroxyl radicals, resulting in the degradation of the dyes [85].

## 6. Other Applications

### 6.1. Wig

Sodium alginate and Antarctic Krill protein combined fiber has similar crystalline structure as human hair. The artificial fiber with superior dyeing performance and unique groove surface proposed practical value for applications in the field of synthetic wig [86].

### 6.2. Diagnosis

Early diagnosis of cancer can increase the survival rate of the cancer patients. Diatrizoic acid (DTA) was conjugated to BSA-based Au nanoparticles through one-pot synthesis process. The nanoparticles showed improved imaging effect of CT on cancer cells, which could be inspected at an earlier stage [87].

Biochips were designed to monitor the antigens and the disease progression. One kind of biochips was specialized for concurrent detection of serum IgG and IgM antibodies against *Borrelia burgdorferi* antigens, flagellin, outer surface protein C, and variable major protein-like sequences in the patients. Recently, the biochip was improved by modifying the gold surface with N-succinimidyl 4-(maleimidomethyl)cyclohexanecarboxylate (SMCC). With enhanced detection ability, the biochip will have a greater potential in clinical applications [88].

### 6.3. Smart Packaging

A mechanically robust and temperature-sensitive biomimetic membrane material based on regenerated silk (RS) nanofibrils was a good candidate of smart packaging. The RS nanofibrils were extracted from the RS solution with high β-sheet content after the single-cell fungi fermentation. As a smart packaging, the biomimetic membrane could intelligently adjust the temperature of the food storage [89].

### 6.4. Atmospheric Water Collection

Achieving efficient atmospheric water harvesting in arid regions is of great significance to the improvement of the local drinking-water supply and quality. Inspired by the *Stenocara* beetle, which could collect water from the moist air in the Namib desert, soy protein-based nanofilm was formed and used to dynamically control the crystal growth of the zeolitic imidazolate framework. After the modification with stearic acid, the micro-/nano-crystals were hydrophobic. And the water from fog was absorbed on the surface of the films [90].

### 6.5. Water Purification

Water is abundant on earth, but fresh water resources are scarce. So the storage of fresh water has been a hot topic of social concern. Membrane separation was the most commonly accepted method to produce fresh water. Living cells-embedded aquaporins (AQPs) showed high water transportation rate. Thus, AQPs were selected as the effective and optional water transport channels in the membrane to increase the rate of water separation and fresh water production [91].

A highly ordered nanoporous multilayer membrane was fabricated through the self-assembly and in situ biomineralization of silk nanofibril (SNF) and hydroxyapatite (HAP), which was a simple and rapid process with low cost. The membrane showed high filter efficiency and high molecular loading capacity and was able to increase water throughput [92].

## 7. Conclusions

This review summarized the formation and application of various functional PBNs and addressed the details of their nanostructures, proteins involved, and synthetic approaches. Table 1 lists the growth factors for tissue regeneration, which can be inserted in the protein-based nanostructures. Table 2 shows various types of protein-based nanoparticles that were applied in targeted anti-cancer drug delivery. Table 3 categorizes the properties and applications of the bioinspired adhesive materials.

Although a search of bioinspired nanoparticles produces about 500 references, which studied the synthesis, characterization, and application of the nanomaterials, few papers reviewed about the protein-based functional nanomaterials application and the relationship among the nanostructures, the functional protein conjunctions, and the applications. The applications of PBNs span a broad range of strategic areas, such as tissue regeneration, precision drug delivery, water purification and collection. So far, although a large amount of new materials were developed, few further mechanisms were known, which may hamper the further developments of the PBNs. We still hold the belief that functional PBNs will have a bright future in the field of medicine, bioengineering, and environment protection.

## 8. Challenges and Future Prospects

A bioinspired system, which mimics the natural components, is versatile and innovative. The development of various bioinspired systems has been conducted in the past several years. The protein-based nanoparticles coupled with the bioactive constituents and functional growth factors are promising approaches for tissue regeneration, precision drug delivery, and environmental engineering.

At present, for treatments of organ failures resulting from injuries, aging, and diseases, surgical transplantation is one of the commonly used strategies. Endogenous tissue regeneration holds enormous promise in overcoming the low availability of acceptable allogenic supplies and postoperative infection of allografts. Designing advanced biomaterials with controlled physical, chemical, electrical, and biological properties will therefore be beneficial to facilitate the formation of the functional tissues.

Targeted cancer therapies offered the potential to improve the treatment of various tumors. The concentration and retention period of anti-tumor agents could be controlled at the site of a tumor, because of the large size and the high loading capacity of the PBNs. The therapeutic agents would be released under controlled conditions, thus, the cytotoxicity to normal cells and tissues might be minimized. The PBNs have provided new insight for the future treatment of cancer.

It is worth to mention that the biomimetic biomaterials are not independent and can be combined together to develop more potent technologies, such as genetic engineering. Molecular biology and functional genomics have been studied for decades, which could be incorporated into new bioinspired materials using synthetic biology tools. Interdisciplinary applications may open up new capabilities in materials design.

## Figures and Tables

**Table 1:** The typical biomaterials used for the various tissue regenerations.

Regenerated Tissues and Cells	Biomaterials Involved	Reference
Bone tissues	collagen	[8,12,13]
	zein	[14,15]
	silk protein	[16,17,18]
Cardiac tissues	silk protein	[19,20]
	Fibrinogen	[21,22]
Corneal tissues	silk protein	[23,24,25,26]
Cartilage tissues	albumin	[27,28]
Vascular tissues	elastin-like peptides	[29,30]
	Fibrinogen	[31,32]
	silk	[33,34]
Nerve tissues	Fibrinogen	[35,36]
	albumin	[37,38]
Skin tissues	gelatin	[39,40]
Muscle tissues	elastin-like peptides	[41,42]

**Table 2:** Types of chemical reactions, substance loading, and conditions that trigger drug release of anti-cancer nanoparticles.

Nanoparticle	Substance Loading	Conditions that Trigger Drug Release	Reference
Albumin	Gemcitabine	NA	[65,66,67,68]
Albumin	Trichosanthin	NA	[69,70]
Albumin	DOX	pH, temperature	[71,72,73,74]
Silk protein	DOX	pH	[75,76,77]

**Table 3:** Adhesive bio-inspired materials application and properties.

Materials	Usage	Property	Reference
mussel foot proteins and CsgA protein	water adhesive	superior adhesion and better tolerance for pH	[82]
hydroxyapatite deposition and polymer substrates	serum protein adsorption	better absorbing of serum protein	[84]
BSA and catalytic hemin-graphene	dyes removement	generation hydroxyl radical	[85]
